
JOURNAL INFORMATION
==============================
NLM Title Abbreviation: JBMR Plus
Journal ID: JBMR Plus
NLM Journal ID: 101707013
Journal ID: jbmrplus

JBMR Plus

EISSN: 2473-4039
Publisher: Oxford University Press

ARTICLE INFORMATION
==============================
PMCID: PMC13710110
DOI: 10.1093/jbmrpl/ziag172
Article ID: ziag172
Article version: 1
Subjects: Abstracts, AcademicSubjects/MED00010, AcademicSubjects/MED00160, AcademicSubjects/MED00250

9th Joint Argentine Congress of Osteology

Publication date: 2026 Nov
Electronic publication date: 2026 Oct 27
Volume: 10
Issue: Suppl 5
First page: v1
Last page: v27
Copyright: © The Author(s) 2026. Published by Oxford University Press on behalf of the American Society for Bone and Mineral Research.
Copyright year: 2026
License: This is an Open Access article distributed under the terms of the Creative Commons Attribution License (https://creativecommons.org/licenses/by/4.0/), which permits unrestricted reuse, distribution, and reproduction in any medium, provided the original work is properly cited.
License URL: https://creativecommons.org/licenses/by/4.0/

Page count: 1

==============================

### 9th Joint Argentine Congress of Osteology

**53rd Annual Scientific Meeting of AAOMM (Argentine Association for Osteology and Mineral Metabolism)**

*and*

**20th Argentine Congress of Osteoporosis (Argentine Society for Osteoporosis – SAO)**

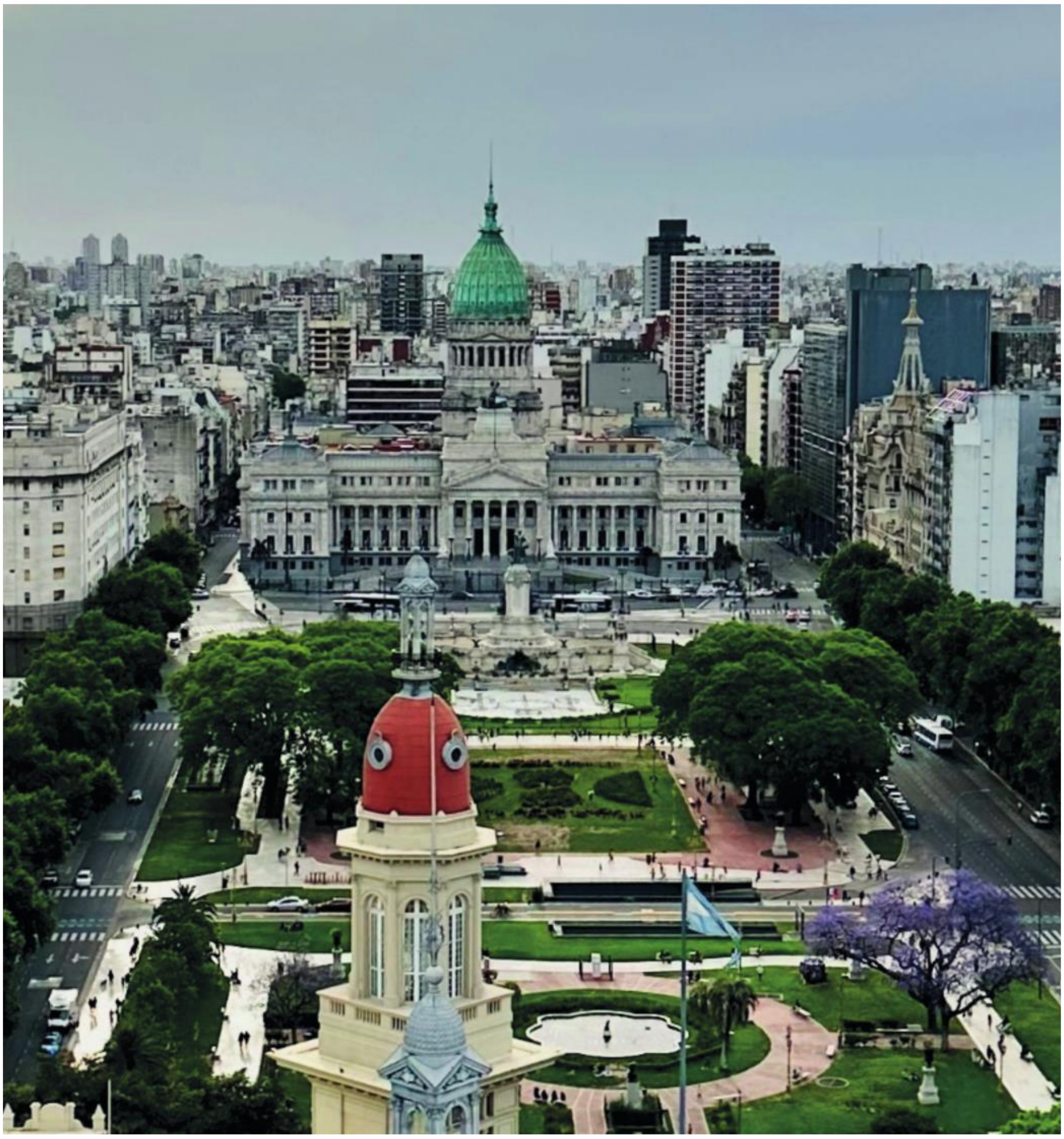

A.A.O.M.M.

**9º CONGRESO ARGENTINO DE OSTEOLOGÍA**

**XLIII Reunión Anual de la AAOMM**

**XX Congreso Argentino de Osteoporosis de SAO**

**5 al 7 de agosto de 2026**

**Palais Rouge - Ciudad Autónoma de Buenos Aires, Argentina**

SAO

### Congress Presidents:

**Antonio Desmond McCarthy**
President of AAOMM
Full Professor and Director of LIOMM
National University of La Plata
La Plata, Argentina

**María Laura García**
President of SAO
Head of Endocrinology
"Julio C. Méndez" Municipal Hospital
Buenos Aires, Argentina

### Scientific Committee:

**Alicia Bagur**
Mautalen Salud e Investigación
Buenos Aires, Argentina

**Pablo Costanzo**
Departamento de Endocrinología
Hospital Italiano de Buenos Aires
Buenos Aires, Argentina

**Vanina Farías**
Instituto de Diagnóstico e Investigaciones Metabólicas (IDIM)
Buenos Aires, Argentina

**Juan Manuel Fernández**
Universidad Nacional de La Plata
Consejo Nacional de Investigaciones Científicas y Técnicas (CONICET)
La Plata, Argentina

**Fabio Massari**
Instituto de Diagnóstico e Investigaciones Metabólicas (IDIM)
Universidad del Salvador
Buenos Aires, Argentina

**María Angélica Rivoira**
Instituto de Investigaciones en Ciencias de la Salud
Universidad Nacional de Córdoba
Córdoba, Argentina

### Organizing Committee:

**Adriana Díaz**
Jefa de Osteopatías, Hospital de Clínicas
Universidad de Buenos Aires
Buenos Aires, Argentina

**Evangelina Giacoia**
Jefa de Endocrinología
Hospital Nacional "Prof. A. Posadas"
Buenos Aires, Argentina

**Diana González**
Mautalen Salud e Investigación
Buenos Aires, Argentina

**Sabrina Lucas**
Sociedad Argentina de Osteoporosis
Buenos Aires, Argentina

**Silvina Mastaglia**
Mautalen Salud e Investigación
Hospital de Clínicas
Universidad de Buenos Aires
Buenos Aires, Argentina

**Paula Rey**
Instituto de Diagnóstico e Investigaciones Metabólicas (IDIM)
Universidad del Salvador
Universidad Católica Argentina
Buenos Aires, Argentina

### Daily summary:

The **9th Joint Argentine Congress of Osteology** (*9º Congreso Argentino de Osteología*) was held at the Palais Rouge venue in Buenos Aires, Argentina, on August 5-7, 2026. Organised jointly by the *Asociación Argentina de Osteología y Metabolismo Mineral* (AAOMM) and the *Sociedad Argentina de Osteoporosis* (SAO), the meeting brought together basic and clinical researchers, endocrinologists, rheumatologists, and bone-health specialists for lectures, interdisciplinary symposia, short-communication presentations, and interactive expert sessions.

### Wednesday, August 5, 2026: Technology, Hormones, Bone Regenerative Biomaterials and microplastics

The congress opened with an inaugural lecture by Dr. Facundo Segura, exploring artificial intelligence applications in osteoporosis management, setting a forward-looking tone for modern bone health diagnostics and therapy. Moving forward, a joint interdisciplinary symposium involving SAO, AAOMM, and the Argentine Society of Andrology (SAA), addressed the role of androgens in skeletal health and bone density maintenance. Small-group *"Meet the Expert"* discussions were also hosted: key practical topics included the application of DXA scans in obese patients and artificial organs in osteo-articular clinical practice. Oral research communications spanned basic and clinical science, covering rare pediatric metabolic bone disorders (such as XLH and HHRH cohort studies) and orthopedic complications. In the afternoon, two symposia were held on cutting-edge topics: *Adipose Tissue and Incretin Therapies* focused on the metabolic intersection of obesity treatments, metabolic surgery, and GLP-1 receptor agonists on bone homeostasis; while *Biomaterials for Bone Regeneration* highlighted clinical translation and tissue engineering, including human-derived therapeutic biomaterials, elastin hydrogels, and antimicrobial hybrid scaffolds for bone repair. The first day closed with a conference by Dr. Rodrigo Bueno de Olivera from Brazil, on the deleterious effects on bone tissue of exposure to dietary and/or environmental micro- and nano-plastics.

### Thursday, August 6, 2026: Novel Anabolics, Bone Infections, and Metabolic Health

The second day opened with oral research communications covering clinical trial outcomes, such as 48-month post-denosumab hip bone mineral density retention via bisphosphonates and public hospital hip-fracture cohort data. In a symposium on unconventional osteoanabolics, experts examined non-standard applications of bone-forming therapies to address challenging skeletal conditions beyond conventional osteoporosis guidelines. Later, in a session on bone infections and microbiome dedicated to bone-pathogen interactions, several experts examined bacterial biofilm adaptations, *Staphylococcus aureus* colonization mechanisms in osteomyelitis, and the systemic effects of HIV infection and antiretroviral therapies on bone turnover. In a symposium examining nutrition beyond calcium and vitamin D, different researchers evaluated micro-architecture and functional foods, presenting studies on bioactive diets, low-lactose formulations with galactooligosaccharides, and nutritional modulators. In another session on bone risk in rheumatological diseases and treatments, experts assessed bone erosion and fracture risk secondary to systemic autoimmune and rheumatologic diseases, alongside tailored therapeutic strategies.

**Friday, August 7, 2026: Plenary Keynote, Sarcopenia, Award-Nominated Research, and Assembly**

In an international keynote lecture, Dr. Martin Blomberg Jensen from Denmark delivered a plenary presentation titled *"Minerals, Bone Factors, and Male Infertility: Clinical Implications?"*, detailing the endocrine feedback mechanisms connecting bone-derived factors and mineral regulators to reproductive health. Moving forward, an interdisciplinary symposium on Sarcopenia updated methods for clinical evaluation of sarcopenia, the effects on skeletal muscle of incretin-based treatments, and the role of RANKL signaling in skeletal muscle function. In interdisciplinary award sessions, top-rated research submissions were presented for congress honors. In the last symposium, the most relevant basic, translational and clinical studies on bone and mineral metabolism published over the past year, were summarized and presented by renowned experts. The Congress concluded with the formal presentation of research awards and the annual AAOMM and SAO institutional assembly meetings.

### Key research studies, clinical topics, and technological innovations:

1. **Clinical Osteoporosis & Pharmacotherapy Findings**

**Post-Denosumab Bisphosphonate Maintenance:** the goal of the study was to determine total hip Bone Mineral Density (BMD) retention 48 months after stopping denosumab. Its key finding was that sequential bisphosphonate therapy successfully preserved bone density gains in a long-term cohort, mitigating the rapid bone turnover rebound typically observed after denosumab discontinuation. **Unconventional Osteoanabolics:** experts reviewed non-standard clinical protocols for osteoanabolic agents (e.g., romosozumab, teriparatide) applied to severe bone fragility cases beyond standard primary osteoporosis guidelines. **Atypical Femoral Fractures (AFF):** long-term registry data examined the incidence, clinical course, and residual skeletal risk in patients who suffered atypical subtrochanteric/femoral shaft fractures secondary to prolonged antiresorptive use.

2. **Metabolic Health, Obesity, and Incretin Therapies**

**GLP-1 Receptor Agonists and Metabolic Surgery:** Symposia assessed the skeletal consequences of modern anti-obesity drugs (GLP-1 receptor agonists like semaglutide) and bariatric surgery. Researchers addressed the trade-off between cardiometabolic benefits and potential loss of lean muscle/bone density. **DXA Interpretation in Severe Obesity:** technical guidelines were presented regarding DXA scanning adjustments and soft-tissue artifact correction for patients undergoing medical or surgical obesity management. **Metformin Protection in Glucocorticoid Osteoporosis:** *in vitro* experimental models demonstrated protective antioxidant and anti-apoptotic effects of metformin against glucocorticoid-induced bone loss and osteoblast damage.

3. **Pediatric & Rare Bone Disorders**

**Pediatric Hypophosphatemic Rickets (HHRH vs. XLH):** in a comparative study, analysis of a pediatric cohort carrying *SLC34A3* variants distinguished the clinical and biochemical phenotypes of Hereditary Hypophosphatemic Rickets with Hypercalciuria (HHRH) from X-linked Hypophosphatemia (XLH). **Orthopedic Quality of Life in Familial Rickets:** a multidisciplinary registry data evaluated orthopedic surgical complications, lower-limb deformities, and functional quality of life scores in children with familial hypophosphatemic rickets treated at tertiary pediatric referral centers. **Hypophosphatasia (HPP):** case series and outcomes from pediatric teams highlighted early enzyme replacement therapy (asfotase alfa) and diagnostic delays in rare metabolic bone diseases.

4. **Infection, Biofilms, and Bone Tissue Engineering**

**Microbial Adaptation in Osteomyelitis:** research highlighted capsule expression dynamics in *Staphylococcus aureus*, showing how phase-variable capsular shifts drive intracellular persistence and deep bone matrix colonization in chronic osteomyelitis. **HIV and Bone Turnover:** presentations evaluated bone mineral loss mechanisms stemming from antiretroviral therapy (ART) regimens combined with HIV-mediated persistent inflammatory signaling. **Translational Regenerative Biomaterials:** experts examined the potential therapeutic applications of human-derived matrices for localized bone defect repair. Additionally, hybrid tissue scaffolds engineered to release targeted antimicrobial agents for prevention of implant-associated osteoarticular infections, were also described and discussed.

5. **Nutrition, Musculoskeletal Aging, and Endocrine Axis**

**Endocrine Connection to Male Fertility (Plenary):** Dr. Martin Blomberg Jensen detailed how bone-derived osteocalcin and vitamin D signaling pathways regulate Leydig cell function and testicular mineral metabolism, linking skeletal status directly to male reproductive health. **Functional Foods & Bioactive Diets:** research presented specialized nutritional formulations—including galactooligosaccharide-enriched low-lactose diets—designed to enhance gut absorption of calcium and preserve micro-architectural bone parameters. **Sarcopenia & Chronic Hemodialysis:** diagnostic criteria using body composition imaging linked nutritional/inflammatory biomarkers to physical frailty and muscle mass reduction in patients undergoing long-term renal replacement therapy.

**TABLE OF CONTENTS:**

9th Joint Argentine Congress of Osteology ........ 1

Congress Presidents ........ 2

Scientific Committee ........ 2

Organizing Committee ........ 3

Daily Summary ........ 4

Key research studies, clinical topics, and technological innovations ........ 5

ABSTRACTS ........ 9

- BEFORE STRUCTURAL DAMAGE: A HIGH-SALT DIET PROMOTES EARLY ADIPOGENESIS AND OSTEOCLASTOGENESIS IN BONE MARROW ........ 9
- NUTRITIONAL, INFLAMMATORY, AND HORMONAL BIOMARKERS: THEIR ASSOCIATION WITH MUSCLE PARAMETERS AND FRAILTY IN CHRONIC HEMODIALYSIS PATIENTS ........ 10
- CORRELATION BETWEEN SONOGRAPHIC FINDINGS AND SEVERITY OF SECONDARY HYPERPARATHYROIDISM IN PATIENTS ON CHRONIC RENAL REPLACEMENT THERAPY: A PILOT STUDY ........ 12
- THE EFFECT OF PROTEIN RESTRICTION AND SUBSEQUENT REINTRODUCTION ON TOOTH ERUPTION IN RATS ........ 13
- BONE DISEASES RESULTING FROM GNAS MUTATION AND ITS ASSOCIATION WITH BREAST CANCER. REGARDING TWO CLINICAL CASES ........ 14
- PRELIMINARY EVALUATION OF OPPORTUNISTIC VERTEBRAL HOUNSFIELD UNITS AND THEIR RELATIONSHIP WITH BONE MINERAL DENSITY, TRABECULAR BONE SCORE, BODY MASS INDEX, AND FRACTURE RISK ........ 15
- THE IMPACT OF FUNCTIONAL FOOD CONSUMPTION ON BONE HEALTH DURING NUTRITIONAL RECOVERY ........ 16
- MICRO-CT AS A TOOL FOR THE EVALUATION OF FUNCTIONAL FOOD MATRICES DURING GROWTH: IMPACT OF A LACTOSE-REDUCED YOGURT AND WITH GALACTOOLIGOSACCHARIDES ON BONE MICROARCHITECTURE ........ 17
- METHOTREXATE-ASSOCIATED OSTEOPATHY IN RHEUMATOID ARTHRITIS PATIENTS: A SERIES OF THREE CASES WITH MULTIFOCAL INSUFFICIENCY FRACTURES OF THE LOWER LIMBS ........ 18
- UTILITY OF THE SARC-F QUESTIONNAIRE AS A COMMUNITY SCREENING: 1 IN 4 OLDER ADULTS AT RISK OF SARCOPENIA ........ 19
- GEOMETRIC, NOT DENSITOMETRIC, ADAPTATION OF THE TIBIAL BONE TO SPORT AND COMPRESSIVE LOADING: A PQCT AND MECHANOSTATIC EFFICIENCY ANALYSIS IN YOUNG MEN ........ 20
- INFLUENCE OF TUNABLE DEGRADATION OF ELASTIN-DERIVED HYDROGELS ON THE REPAIR OF OSTEOCHONDRAL LESIONS ........ 21

DESCRIPTIVE ANALYSIS OF A HIP FRACTURE COHORT IN A PUBLIC HOSPITAL IN BUENOS AIRES: GAPS IN SECONDARY OSTEOPOROSIS PREVENTION ………. 22

IMPACT OF SUBCUTANEOUS SEMAGLUTIDE AND TIRZEPATIDE ON APPENDICULAR MUSCLE MASS: A PROSPECTIVE REAL-WORLD COHORT ASSESSED BY DXA .. 23

IMPACT ON THE QUALITY OF LIFE IN PATIENTS DIAGNOSED WITH MEDICATION RELATED OSTEONECROSIS OF THE JAW (MRONJ) IN THE ONLY SPECIALIZED DENTAL CENTER IN ARGENTINA ……………………………………………………………… 24

INCIDENCE, CLINICAL OUTCOMES AND LONG-TERM SKELETAL RISK IN A COHORT OF PATIENTS WITH ATYPICAL FEMORAL FRACTURE ……………………………….. 25

UTILITY OF TRABECULAR BONE SCORE FOR IDENTIFYING VERTEBRAL FRACTURES IN RHEUMATOID ARTHRITIS: EVIDENCE FROM AN ARGENTINE COHORT … 26

### ABSTRACTS:

### BEFORE STRUCTURAL DAMAGE: A HIGH-SALT DIET PROMOTES EARLY ADIPOGENESIS AND OSTEOCLASTOGENESIS IN BONE MARROW

**Quiroga C (1), Acosta Rodríguez EV (3), Rivoira MA (1)**

*(1) Instituto de Investigaciones en Ciencias de la Salud (CONICET). (2) Centro de Investigación en Bioquímica Clínica e Inmunología (CONICET). Córdoba, Argentina.*

A high-salt diet (HSD) is a risk factor for bone health. Besides increasing urinary calcium excretion, sodium may affect bone remodeling through immune-mediated mechanisms. We previously reported that, in uninephrectomized (UNX) mice, 20 days of HSD (4% NaCl) induces early pro-inflammatory immune activation in bone marrow, without detectable changes in bone structure or biomechanics. This study evaluated the early effects of HSD on bone cells and other bone marrow niche cells. Male and female Foxp3GFP mice (7–10 weeks old, n=3 of each/group) underwent UNX and, after recovery, received either a normal-salt diet (NSD, 0.2% NaCl) or HSD (4% NaCl) for 20 days. After, tibias were measured and processed for histological analysis using hematoxylin-eosin and tartrate-resistant acid phosphatase staining. Growth plate height and the densities of osteocytes, adipocytes, megakaryocytes, and osteoclasts were quantified. Data are expressed as mean±SD and compared using Student's t-test. Tibial length and growth plate height did not differ between groups (17.2±0.2 vs 17.2±0.3 mm, p=0.89; 424.8±35.5 vs 405.9±30.9 µm, p=0.32, respectively). In contrast, HSD significantly increased adipocyte (4.8±2.5 vs 1.4±0.8 cells/mm², p=0.02) and osteoclast density (0.9±0.3 vs 0.6±0.1 cells/mm², p=0.04) compared with NSD. Megakaryocyte and osteocyte density were unchanged. These findings demonstrate that short-term HSD intake alters the bone marrow microenvironment before evident bone damage develops. The early response is characterized by increased adipogenesis and osteoclastogenesis, suggesting that sodium-related inflammatory changes may contribute to subsequent bone deterioration.

**NUTRITIONAL, INFLAMMATORY, AND HORMONAL BIOMARKERS: THEIR ASSOCIATION WITH MUSCLE PARAMETERS AND FRAILTY IN CHRONIC HEMODIALYSIS PATIENTS.**

**Moine LB (1,2), Quiroga C (1,2), Flores Corregidor I (1), Ariaudo D (3), Olmedo MB (3), Porta D (4), Olmos JM (3), Novoa P (3), Rivoira MA (1,2).**

*(1) Cátedra de Bioquímica y Biología Molecular, Facultad de Ciencias Médicas, UNC. (2) Instituto de Investigaciones en Ciencias de la Salud (INICSA-CONICET). (3) Servicio de Nefrología, Sanatorio Allende, Córdoba. (4) Centro de Investigación en Bioquímica Clínica e Inmunología (CIBICI-CONICET).*

Introduction: Patients with chronic kidney disease (CKD) on hemodialysis (HD) frequently develop malnutrition, muscle wasting, and frailty, increasing morbidity. Objective: To evaluate nutritional status, dietary intake, muscle function, and biochemical parameters in HD patients and explore their association with frailty. Materials and methods: Cross-sectional study in 31 HD patients. Dietary intake, body composition (BIA), handgrip strength (HGS), and physical performance (SPPB) were assessed. Serum albumin (ALB), phosphorus, creatinine, CPK, hs-CRP, sDHEA, and vitamin D were measured. Pearson's correlation and multiple linear regression (MLR) were performed. Results: Participants were 61.3% male (27–90 years); diabetic nephropathy was the main cause of CKD (29%). Excess weight was observed in 61.3% of patients. High body fat was present in >80%, while reduced muscle mass affected 58.8% of men and 50% of women. Adequate energy and protein intake were achieved by 42% and 58%, respectively. Low HGS was detected in 10.5% of men and 36.4% of women. Frailty was identified in 19.4% and risk of frailty in 61.3%. ALB was normal in 96.8% (4.02±0.28g/dL). MLR showed a trend toward an inverse association between serum phosphorus and HGS (ß=-2.57; p=0.085). Conclusion: HD patients exhibited impaired muscle mass and function with high frailty prevalence despite normal ALB. sDHEA, hs-CRP, and ALB may be useful biomarkers of muscle risk. Phosphate control deserves further investigation regarding muscle impairment.

Table 1. Pearson correlation analysis.

| Variables | p | R |
|---|---|---|
| sDHEA | | |
| Alb | 0,001 | 0,55 |
| Muscle mass | 0,010 | 0,50 |
| Dynamometry | 0,003 | 0,52 |
| Alb | | |
| SPPB score | 0,040 | 0,35 |
| Muscle mass | 0,020 | 0,40 |
| Walking time | 0,020 | -0,40 |
| PCRus | | |
| Alb | 0,030 | -0,40 |
| sDHEA | 0,030 | -0,39 |
| Vitamin D | 0,005 | -0,51 |
| Muscle mass | | |
| Dynamometry | 0,030 | 0,40 |
| Animal protein intake | 0,040 | 0,36 |
| BMI | 0,040 | -0,37 |
| Body fat | 0,0001 | -0,89 |
| Walking time | 0,020 | -0,40 |
| Body fat | | |
| Alb | 0,030 | -0,38 |
| Vitamin D | 0,030 | -0,40 |
| Protein intake | 0,003 | -0,52 |
| BMI | 0,0002 | 0,63 |
| Walking time | 0,020 | 0,41 |

### CORRELATION BETWEEN SONOGRAPHIC FINDINGS AND SEVERITY OF SECONDARY HYPERPARATHYROIDISM IN PATIENTS ON CHRONIC RENAL REPLACEMENT THERAPY: A PILOT STUDY

**Pincay, I; Paz Wasiuchnik, V; Herbas, P**

*Hospital Donación Francisco Santojanni, Buenos Aires, Argentina.*

Introduction: Secondary hyperparathyroidism (SHPT) in chronic kidney disease (CKD) arises from inadequate parathyroid hormone (PTH) secretion. Cervical ultrasound is the preferred initial imaging modality, with a reported sensitivity of 93%. Objective: Sonographic detectability, number, size, morphology, and vascular pattern of parathyroid glands (PTG) are associated with SHPT severity. Methods: 18 dialysis patients (15 hemodialysis, 3 peritoneal dialysis) were evaluated between April and May 2026. PTH measured by electrochemiluminescence (Cobas Pro E801, pg/mL). Ultrasound was performed by a single operator with SonoSite equipment. The echostructural patterns of PTG were classified as: hypoechoic, mildly heterogeneous, highly heterogeneous, and hyperechoic. When the gland showed ultrasonographic alterations, it was classified as highly heterogeneous. Each visible parathyroid gland was measured, and the individual and total glandular volumes were calculated using the ellipsoid formula. Based on the color and power Doppler signal detected, vascularization was classified as: C1, central flow <30%; C2, central flow 30-50%; C3, central flow >50%. A1 arc flow <50%; A2 arc flow >50%; Mild mixed C1-C2 + A1; High mixed C3+A2. Results: Multiglandular detectability (visible PTGs) was the only significant predictor of SHPT severity (Spearman $\rho=0.551$, $p=0.018$), with limited post hoc power. More detected glands correlated with higher PTH, consistent with multiglandular hyperplasia in end-stage CKD. No significant correlations between PTH/Ca×P and volume, echostructure, or Doppler flow. Conclusions: Multiglandular detectability may predict the severity of SHPT. These findings require validation with a higher n.

**THE EFFECT OF PROTEIN RESTRICTION AND SUBSEQUENT REINTRODUCTION ON TOOTH ERUPTION IN RATS.**

**Bozal CB (1), Biagini S (1), Sánchez LM (1), Zeni Coronel M (2), Zeni S (2), Seijo M (2).**

*(1) Cátedra de Histología y Embriología, Facultad de Odontología, Universidad de Buenos Aires (UBA). (2) Laboratorio de Osteopatías Metabólicas, INIGEM/CONICET/UBA. Buenos Aires, Argentina.*

Protein restriction during early life can alter craniofacial skeletal growth. Nutritional recovery promotes compensatory growth developmental parameters. The objective was to analyze the impact of protein restriction and nutritional recovery through protein repletion on tooth eruption and the formation of the developing dental alveolus in growing rats. Weaned male rats (n=24) were divided into two experimental groups. During the first 7 days post-weaning, the control group (C) received an AIN-93G growth diet, while the protein-restricted group (R) was fed a modified AIN-93G diet containing 4% protein to induce protein malnutrition. From day 8 until the end of the study (day 28), both groups were fed the AIN-93G diet. Jaws were collected post-euthanasia for histomorphometric and histological analysis. Using microphotographs of buccolingual sections taken at the level of the mesial root of the first molar, the degree of tooth eruption (DE), root length (RL), length of the vestibular (VL) and lingual (LL) tables, and the area of the developing alveolar base were determined. At 7 days post-experiment, the R group exhibited a significantly greater LV than the C group (1899.19 ± 65.40 µm vs. 1781.17 ± 55.53 µm*). In contrast, the alveolar base area and LR were significantly lower in group R compared to group C (103.47 µm² vs. 1690.06 ± 53.09 µm²* and 1690.06 ± 53.09 µm vs. 1849.29 ± 86.53 µm*, respectively). At the end of the nutritional recovery period (28 days), no significant differences were observed between the two groups for any of the evaluated parameters. Dentoalveolar developmental deficits caused by protein restriction are fully reversible with subsequent protein replenishment.

**BONE DISEASES RESULTING FROM GNAS MUTATION AND ITS ASSOCIATION WITH BREAST CANCER. REGARDING TWO CLINICAL CASES.**

**Mastaglia SR**

*Laboratorio de Osteoporosis y Enfermedades Metabólicas Óseas. Instituto de Inmunología, Genética y Metabolismo (INIGEM-CONICET), Hospital de Clínicas, Universidad de Buenos Aires. Buenos Aires, Argentina.*

Background: The mutation in Gsα-coding GNAS exons is the most frequent mutation across all heterotrimeric G proteins involved in oncogenesis[1]. Objective: To describe the clinical cases of two patients with bone disease diagnosis resulting from GNAS mutation who developed breast cancer[1]. Clinical case #1: *Fibrous* dysplasia *(FD):* A 33-year-old woman with McCune-Albright syndrome (MAS) diagnosed at age 7 due to precocious puberty and polyostotic FD. At age 29, a nodule in the left breast was detected during routine gynecological examination. Partial mastectomy of the left breast was performed, followed by 39 sessions of adjuvant radiotherapy. The histopathological examination showed juvenile papillomatosis with foci of intraductal carcinoma, and immunolabeling studies showed a 90% estrogen receptor positive and a 15% progesterone receptor positive status. She underwent tamoxifen therapy for 5 years. Clinical case #2: *Pseudohypoparathyroidism (PHP):* A 42-year-old woman with PHP diagnosed at age 8 [parathyroid hormone (PTH): 416 pg/ml (reference value (rv): 15-65)] with a family history (sister). At the time of the first evaluation, the CT scan showed symmetric basal ganglia calcifications, and renal ultrasound revealed a kidney stone of 3.4 mm. The patient received the standard treatment for PHP with an adequate response. At age 39, a nodule in the left breast was detected during routine gynecological examination. Partial mastectomy of the left breast and axillary lymph nodule dissection ($25/4^+$) was performed, followed by chemotherapy, radiotherapy, and hormone therapy (anastrozole/leuprolide acetate). The histopathological examination showed infiltrating ductal carcinoma with high-grade intraductal component. Conclusion: From a clinical perspective, these two cases of breast cancer in association with FD/MAS and PHP show the importance of emphasizing routine gynecological examination in patients with bone diseases resulting from GNAS mutation.

[1]Raut P, *et.al*. *Contract to kill: GNAS mutation*. Molecular Cancer (2025) 24:70

**PRELIMINARY EVALUATION OF OPPORTUNISTIC VERTEBRAL HOUNSFIELD UNITS AND THEIR RELATIONSHIP WITH BONE MINERAL DENSITY, TRABECULAR BONE SCORE, BODY MASS INDEX, AND FRACTURE RISK**

**Larroudé MS, Sarnagiotto P, Contreras J.**

*Centro Rossi, Buenos Aires, Argentina.*

Objective: To evaluate the association between vertebral HU opportunistic CT and BMD, body mass index (BMI), Trabecular Bone Score (TBS), and FRAX. Materials and Methods: A cross-sectional study was conducted including 38 postmenopausal women who underwent DXA and opportunistic vertebral HU measurements on CT scans. Demographic, anthropometric, densitometric, and clinical variables were collected, including age, BMI, BMD, T-score, TBS, FRAX major osteoporotic fracture, and FRAX hip fracture risk. Clasification HU: <100 (high probability of osteoporosis), 100–160 (intermediate risk of low bone mass), and >160 (low probability of osteoporosis). Stadistic: mean ± standard deviation, Pearson correlation coefficients and analysis of variance (ANOVA). Statistical significance was established at $p < 0.05$. Results: Thirty-eight women were included, mean age of 61.4 years and a BMI of 27.5 kg/m², vertebral attenuation was 121.4 ± 46.0 HU. HU demonstrated a moderately strong positive correlation with BMD ($r = 0.68$) and T-score ($r = 0.70$), indicating that higher BMD values were associated with higher HU values. Negative correlations between HU and age ($r = -0.36$), FRAX major osteoporotic fracture risk ($r = -0.37$), and FRAX hip fracture risk ($r = -0.38$), suggesting that lower HU values were associated with increased fracture risk. The correlation HU and BMI was weak ($r = 0.20$). Mean HU values differed significantly according to DXA classification, with values of 149.4 HU in patients with normal BMD, 99.3 HU in those with osteopenia, and 83.8 HU in those with osteoporosis (ANOVA $p = 0.0005$). Among overweight and obese patients (BMI ≥25 kg/m²), 77.3% had HU values <160 and 27.3% had HU values <100. Pa tients with normal BMD and BMI ≥25 kg/m², 61.5% exhibited HU values <160 and 7.7% had HU values <100.TBS data were available in 11 patients. TBS showed a moderate correlation with BMD ($r = 0.54$) and T-score ($r = 0.54$), but no correlation with HU ($r = 0.01$). Conclusions: These preliminary findings support the complementary role of vertebral HU assessment in the evaluation of bone health and fracture risk.

**THE IMPACT OF FUNCTIONAL FOOD CONSUMPTION ON BONE HEALTH DURING NUTRITIONAL RECOVERY**

**Mascias H (1), Palomino I (1), Zeni Coronel EM (1), Katz ME (1), Bozal CB (2), Rosetti F (1), Zeni S (1), Seijo M (1).**

*(1) INIGEM/CONICET/UBA. (2) Department of Histology and Embryology, FOUBA. Buenos Aires, Argentina.*

Protein malnutrition during growth impairs physical development and bone mass acquisition. Prebiotics may promote nutritional recovery. The objective was to evaluate the effect of galacto-oligosaccharide (GOS) supplementation on bone mass acquisition in an experimental model of post-protein-malnutrition nutritional recovery. During the first 7 days, 24 male Wistar rats were divided into a control group (C), fed an AIN-93G diet, and a protein-restricted group (R), fed an AIN-93G diet modified with 4% protein to induce malnutrition. From day 8 through day 29, the animals were subdivided into four groups: CC: fed AIN-93G; RC: previously malnourished animals fed AIN-93G; RP: previously malnourished animals fed AIN-93G supplemented with GOS. Were evaluated: food intake (g/day), body length (BL; cm), body weight (BW; g), cecal weight (C; g/100 g BW), *Lactobacillus spp*. count (LCC; Log CFU/g feces), bone volume (BV; %), total cartilage (GPC.th; µm) and hypertrophic cartilage (HpZ.th; µm). At 7 days post-experiment, the R group exhibited lower feed intake, BW, BV, GPC.th and HpZ.th compared to group C. No differences were observed in LC, C, or RCL between groups. At the end of the study, the RC group showed lower BW, LCC, BV, GPC.th, and HpZ.th than CC. In contrast, the RP group exhibited higher BW, LCC, BV, GPC.th, and HpZ.th than RC, with no differences in feed intake, LC, or C. Moreover, BW, BV, GPC.th, and HpZ.th in RP reached values comparable to CC, while LCC was significantly higher. In this model GOS supplementation improved parameters associated with intestinal fermentation and bone, supporting a nutritional strategy to optimize bone mass acquisition under adverse conditions during growth.

**MICRO-CT AS A TOOL FOR THE EVALUATION OF FUNCTIONAL FOOD MATRICES DURING GROWTH: IMPACT OF A LACTOSE-REDUCED YOGURT AND WITH GALACTOOLIGOSACCHARIDES ON BONE MICROARCHITECTURE**

**Mascias H (1), Zeni Coronel EM (1), Vénica C (2), Longo M (1), Vazquez D (3), Gomez A (3), Perotti MC (2), Zeni S (1), Seijo M (1).**

*(1) INIGEM/CONICET/UBA. (2) INLAIN, UNL/CONICET. (3) FOUBA. Buenos Aires (1,3) y Santa Fe (2), Argentina.*

Galactooligosaccharides (GOS) are prebiotic compounds derived from lactose that may enhance mineral absorption through modulation of the intestinal microbiota. The present study evaluated the usefulness of micro-computed tomography (microCT) for assessing the impact of functional food matrices on bone quality during normal growth, using an experimental yogurt naturally reduced in lactose and containing GOS (YE) as a study model. Recently weaned male rats (n=8/group) were fed for 30 days with either a standard AIN-93G diet (C), a diet based on regular yogurt without GOS (YR), or a diet formulated with the experimental yogurt (YE). Trabecular bone microarchitecture of the distal femur was analyzed by microCT, including bone volume fraction (BV/TV), bone surface density (BS/TV), trabecular thickness (Tb.Th), trabecular number (Tb.N), trabecular separation (Tb.Sp), and connectivity density (Conn.D). MicroCT analysis revealed a significantly higher BV/TV in the YE group compared with both C and YR groups ($p<0.05$). In addition, YE showed higher BS/TV, Tb.Th, Tb.N, and Conn.D values, together with lower Tb.Sp values, indicating a trend toward improved trabecular microarchitecture, although these differences did not reach statistical significance. In conclusion, microCT proved to be a sensitive tool for evaluating functional food matrices in experimental models of growth. Consumption of the GOS-containing lactose-reduced yogurt improved bone quality parameters, mainly through a significant increase in BV/TV, highlighting its potential as a nutritional strategy to support bone mass acquisition during growth.

**METHOTREXATE-ASSOCIATED OSTEOPATHY IN RHEUMATOID ARTHRITIS PATIENTS: A SERIES OF THREE CASES WITH MULTIFOCAL INSUFFICIENCY FRACTURES OF THE LOWER LIMBS**

**Guelman R, Aliquo N, Collaud C, González V, Sabelli M, Scolnik M**

Metabolic Bone Disease, Nuclear Medicine (Endocrinology & Nuclear Medicine Dept.), Rheumatology Section, Hospital Italiano de Buenos Aires. Buenos Aires, Argentina

Introduction: Methotrexate (MTX)-associated osteopathy is a rare and likely underdiagnosed complication characterized by bone pain and insufficiency fractures, mainly in the lower limbs, which may be mistaken for RA-related osteoporosis or typical osteoporotic fractures. Objective: To describe the clinical, imaging, and follow-up features of three RA patients with recurrent insufficiency fractures compatible with MTX-associated osteopathy. Methods: Retrospective review of three RA patients on long-term MTX referred for multiple stress fractures, analyzing fracture location, imaging findings, anti-osteoporotic treatments, and outcomes after MTX discontinuation. Results: Three women aged 55, 69, and 76 with long-standing RA and prolonged MTX exposure (15–25 mg/week) presented with progressive lower limb pain and multiple bilateral, multifocal insufficiency fractures. Sites included femoral metaphyses, tibial plateaus, calcanei, and metatarsals. In two patients, fractures persisted despite teriparatide, denosumab, and/or bisphosphonates. MRI identified multifocal stress fractures in all cases; bone scintigraphy with SPECT/CT demonstrated extensive bilateral skeletal involvement in one patient. After MTX withdrawal and switch to alternative DMARDs, all three showed clinical improvement and no new fractures. Conclusions: MTX-associated osteopathy should be considered in RA patients with mechanical pain and recurrent insufficiency fractures, especially when bilateral, multifocal, or disproportionate to bone mineral density. MRI is the diagnostic method of choice; MTX discontinuation appears central to favorable outcomes.

**UTILITY OF THE SARC-F QUESTIONNAIRE AS A COMMUNITY SCREENING: 1 IN 4 OLDER ADULTS AT RISK OF SARCOPENIA**

**Farias VS, Garcia ML, Elias N, Cowan MP, Rosmino J, Pissani MP, Torrecilla I**

*Sociedad Argentina de Osteoporosis. Buenos Aires, Argentina.*

Introduction: Sarcopenia is highly prevalent in older adults and associated with falls, frailty and increased mortality. The SARC-F questionnaire (EWGSOP2, 2019) is recommended as a simple, validated screening tool applicable at any care level. Objective: To estimate the prevalence of sarcopenia risk using the SARC-F in community-dwelling older adults attending health education talks. Methods: Cross-sectional descriptive study. The SARC-F was self-administered during two community talks. Adults ≥60 years with complete questionnaire were included (n=73). Anthropometric data (age, weight, height, BMI) and sex were recorded. A score ≥4 was defined as probable sarcopenia risk (EWGSOP2). Frequencies, percentages, means and ranges were calculated. Results: 73 participants (94.5% women; mean age 70 years; mean BMI 26.8 kg/m$^2$; and 24.7% (18/73) scored ≥4 (sarcopenia risk). Positivity peaked in the 75–79 age group (42.9%; 6/14). Most affected domains: stair climbing (43.8%), carrying weight (41.1%) and rising from a chair (38.4%). 32.9% reported ≥1 fall in the past year. Conclusion: 1 in 4 community-dwelling older adults screened positive for probable sarcopenia. The SARC-F proved feasible and effective in a community setting, supporting its systematic implementation as an early detection strategy.

### GEOMETRIC, NOT DENSITOMETRIC, ADAPTATION OF THE TIBIAL BONE TO SPORT AND COMPRESSIVE LOADING: A PQCT AND MECHANOSTATIC EFFICIENCY ANALYSIS IN YOUNG MEN

**<u>Cointry G</u>, Pisani L, Intelangelo L, Mildenberger C, Lutterotti M, Aloni L, Costamagna P, Luscher S, Capozza R**

*Centro de Estudios de Metabolismo Fosfocálcico, CEMFoC, Universidad Nacional de Rosario, Rosario, Argentina; Unidad de Investigación Musculoesquelética (UIM), Centro Universitario de Asistencia,Docencia e Investigación (CUADI), Universidad del Gran Rosario, Rosario, Argentina; Unidad de Estudios Biomecánicos Osteomusculares (UDEBOM), Centro Universitario de Asistencia, Docencia e Investigación (CUADI), Universidad del Gran Rosario, Rosario.*

Introduction and objectives. The mechanostat theory posits that bone adapts its design by redistributing material according to habitual loads, rather than by changing tissue density. We aimed to characterize the structural differences of the tibia associated with distinct sport loading regimens —integrating the trabecular compartment, diaphyseal geometry and mechanostatic efficiency— and to assess the relationship between compressive stimulus and trabecular bone at the individual level. Methods. Cross-sectional study in 79 young men: football (n=31), runners (n=10), rugby (n=23), swimming (n=5) and sedentary controls (n=10). Serial pQCT scans were obtained every 5% of tibial length; cortical/trabecular separation used an automatic concentric peeling algorithm. Trabecular density (TbD), area (TbA) and content (TbC), polar moment of inertia (CSMIp) as diaphyseal geometric robustness, and BSIap (=anteroposterior moment × cortical density) were measured. EMrot=BSIap/Frot and EMvec=BSIap/Fplat were computed, with Fplat (jump, kN) and Frot (ankle eversion, kg-force). Statistical analysis: per-slice ANCOVA (group factor; covariates age, weight, height; sedentary reference) with adjusted marginal means; individual partial correlations force-trabecular; exploratory slice-by-slice analysis. Significance p<0.05. Results. Trabecular density did not differ between groups (distal p=0.58; proximal p=0.17). Diaphyseal CSMIp showed the most consistent group effect (p=0.019), persisting after adjustment with weight non-significant (p=0.40); rugby, football and runners exceeded swimming and sedentary. Reinforcement was topographically localized: distal (10-20%) in runners, diaphyseal (40-75%) in rugby and football; swimming did not differ from sedentary (limited power, n=5). EMrot discriminated at 3 slices (15,20,40%) and EMvec at 1 (20%); force-adjusted BSIap retained a group effect (p=0.03), ruling out a denominator artifact. Individually, jump force (compressive stimulus) was associated with trabecular amount (TbA, TbC) but not density (TbD), independently of sport. Conclusions. Sport-associated tibial bone differences are expressed through geometric redistribution of material — both cortical and trabecular— and not through density changes, with a discipline-specific topographic signature. The individual association between compressive stimulus and trabecular amount (not density) directly illustrates the compressive function of the mechanostat. EMrot and EMvec are useful for non-invasively exploring the functional quality of bone design.

**INFLUENCE OF TUNABLE DEGRADATION OF ELASTIN-DERIVED HYDROGELS ON THE REPAIR OF OSTEOCHONDRAL LESIONS**

**Juanes-Gusano MD, Puertas-Bartolomé DM, Sabariz Z, Alvarez JL, Francioni N, Orellana MF, Operti C, Sarrió L, Mardegan Issa JP, Stur M, Alonso M, Rodríguez-Cabello JC, Feldman S**

*BIOFORGE, Spain; LABOATEM, Argentina; Ribeirão Preto School of Dentistry, Brazil; Dep. Diagnostic Imaging, Argentina; Central Hospital Mendoza, Argentina*

We evaluated the effect of degradation kinetics of elastin-like recombinamer (ELR) hydrogels on osteochondral repair. ELRs containing RGD cell-adhesion motifs and protease-sensitive domains, DRIR (slow-degrading) and GTAR (fast-degrading), were produced by click chemistry and characterized by SDS-PAGE after enzymatic degradation. Cytocompatibility and chondrocyte proliferation were assessed in vitro. Twenty rabbits were assigned to four groups (n=5): healthy controls, untreated osteochondral lesions, lesions treated with RGD-DRIR, and lesions treated with RGD-GTAR. Clinical, hematological, MRI, and histopathological evaluations were performed. GTAR-containing ELRs degraded faster than DRIR-containing ELRs. Both hydrogels showed cytocompatibility, while chondrocyte proliferation was significantly greater with RGD-GTAR ($p<0.01$). No significant clinical or hematological differences were observed among groups. MRI at 90 days demonstrated cartilage formation in both treated groups, whereas untreated lesions showed no repair. Histology revealed sparse cartilage and low chondrocyte density in untreated defects. RGD-DRIR increased chondrocyte numbers but generated disorganized tissue with limited extracellular matrix. RGD-GTAR promoted organized cartilage with isogenous chondrocyte groups and an appropriate extracellular matrix-to-cell ratio. Conclusion: Fast-degrading ELR hydrogels enhanced cartilage organization, highlighting degradation kinetics as critical for osteochondral repair.

### DESCRIPTIVE ANALYSIS OF A HIP FRACTURE COHORT IN A PUBLIC HOSPITAL IN BUENOS AIRES: GAPS IN SECONDARY OSTEOPOROSIS PREVENTION

**Sales S, Edber C, Planas Y, Rocha V, Romero C, Ruibal G, Suárez M, Schwartz N.**

*Endocrinology Unit, Hospital T. Álvarez, Buenos Aires, Argentina.*

Introduction: Hip fracture (HF) is a serious osteoporosis complication, with high mortality and refracture risk; post-fracture liaison services are scarce in Argentine public hospitals. Objective: to describe clinical, biochemical and care-trajectory features of HF patients, focusing on pre- and post-fracture care gaps, as a baseline for an institutional pre-discharge zoledronate (ZLD) program recently implemented. Methods: observational, descriptive, retrospective study with post-discharge telephone follow-up (recall) in adults admitted for fragility HF (March 2024–April 2026) in a public hospital in Buenos Aires. ZLD eligibility per Johansen 2023 (creatinine clearance >30 mL/min, no hypocalcemia). Continuous variables: median [IQR] or mean±SD; categorical: n (%). Results:48 patients, 41 (85.4%) women; median age 81.0 [74.5–86.0] years. Only 5 (10.4%) had received prior antiosteoporotic treatment (AOT) despite 13 (27.1%) having a prior fragility fracture; in this subgroup (n=13), 10 (76.9%) were never treated. Fall-predisposing drugs 19 (39.6%); insufficient dietary calcium 37/46 (80.4%). 25(OH)D 17.0 [9.8–26.0] ng/mL. ZLD eligibility 36/41 (87.8%). Recall: follow-up available in 20/48 (41.7%); mortality 4 (20%); new fractures 5 (25%); specialist consultation 9 (45%). Current AOT (n=12): on treatment and adherent 6 (50%); zoledronate was the most indicated drug (70%). Conclusion: most patients reached HF without AOT despite sentinel events, and follow-up confirmed refractures, mortality and poor adherence: a prevention circuit broken at both ends. These data are the starting point of the ongoing implementation of the first institutional pre-discharge ZLD protocol.

**IMPACT OF SUBCUTANEOUS SEMAGLUTIDE AND TIRZEPATIDE ON APPENDICULAR MUSCLE MASS: A PROSPECTIVE REAL-WORLD COHORT ASSESSED BY DXA**

**Di Gregorio S, Brance ML, Brun LR, Nasazzi Doddi P, Ramírez Stieben LA**

*Unidad Cardio-Endocrino-Metabólica, Centre Mèdic Vic, Barcelona, Spain. Reumatología y Enfermedades Óseas y Laboratorio de Biología Ósea UNR, Rosario, Argentina.*

Introduction and objectives: In patients with obesity and/or diabetes, weight loss induced by incretin-based therapies may be associated with heterogeneous changes in body composition. From an osteometabolic perspective, characterizing the partitioning of weight loss is clinically relevant, as reductions in muscle mass may affect strength, physical function, frailty, and the risk of sarcopenia. The aim of this study was to evaluate changes in total lean mass, appendicular lean mass (ALM), and appendicular lean mass index (ALMI) during treatment with subcutaneous semaglutide or tirzepatide, compare these changes between therapies, and analyze their relationship with the magnitude of weight loss. Materials and methods: This was a prospective, observational, real-world study of adults treated with subcutaneous semaglutide or tirzepatide for obesity and/or diabetes at the Cardio-Endocrine-Metabolic Unit of Centre Mèdic Vic, Barcelona, Spain. Patients with two DXA body composition assessments were included. The main analysis comprised 118 subjects exposed to incretin-based therapies. Within-subject changes between scans were assessed using paired tests. Between-treatment differences were analyzed both unadjusted and using multivariable linear models adjusted for age, sex, diabetes, previous treatment, dose, baseline BMI, baseline value of the dependent variable, and time between scans. The relationship between weight loss and changes in ALM and ALMI was also explored. Results: A total of 118 patients receiving incretin-based therapies were analyzed, 63 treated with subcutaneous semaglutide and 55 with tirzepatide; 73.5% were women and the median age was 57.2 years. Regarding weight-loss response, 83.1% of patients achieved ≥5% weight loss and 44.1% achieved ≥10% weight loss. Between the two DXA assessments, significant reductions were observed in total lean mass [Δ -2.18 kg (IQR -3.67 to -0.75); p<0.001], appendicular lean mass (ALM) [Δ -0.86 kg (IQR -2.01 to -0.20); p<0.001], and ALMI [Δ -0.30 kg/m² (IQR -0.76 to -0.07); p<0.001]. Approximately one quarter of total weight loss was attributable to total lean mass loss, and 11.1% to appendicular lean mass loss. In the unadjusted analysis, no significant differences were observed between semaglutide and tirzepatide for ΔALM (p=0.124) or ΔALMI (p=0.063). In multivariable models, treatment type was not independently associated with changes in ALM or ALMI. In addition, the magnitude of weight loss correlated significantly with reductions in ALM (rho=0.611; p<0.001) and ALMI (rho=0.614; p<0.001), with greater absolute losses observed as weight loss increased. No evidence was found that the association between the magnitude of weight loss and change in ALM differed between subcutaneous semaglutide and tirzepatide. Conclusions. In this real-world cohort, subcutaneous semaglutide and tirzepatide were associated with a significant reduction in DXA-estimated muscle mass, occurring in parallel with predominantly fat loss. The magnitude of muscle loss increased with greater weight reduction, with no independent differences between the two therapies. These findings reinforce the value of body composition assessment during incretin-based treatment and support strategies aimed at preserving skeletal muscle in patients at risk of sarcopenia or frailty.

**IMPACT ON THE QUALITY OF LIFE IN PATIENTS DIAGNOSED WITH MEDICATION RELATED OSTEONECROSIS OF THE JAW (MRONJ) IN THE ONLY SPECIALIZED DENTAL CENTER IN ARGENTINA.**

**Picardo SN, Rodriguez Genta SA, Castiglia VC.**

*Dental Center specializing in MRONJ, CABA, Argentina.*

Introduction: MRONJ is a rare contemporary condition, its diagnosis and treatment should be handled by a dentist. Objective: To evaluate the impact on the quality of life of patients involved in: “Retrospective study of patients diagnosed with Medication Related Osteonecrosis of the Jaw (MRONJ) were surgically treated” as a sample of the partial results of the Doctoral Thesis of PhDs. Sergio A. Rodriguez Genta, Pontifical Catholic University of Argentina. The data were collected in CABA, Argentina, between July 8, 2024 and July 8, 2025. Materials and Methods: A survey on quality of life during the course of MRONJ was sent by WhatsApp, assessing: perception of symptoms, disruption to daily routine, clinical recovery, and impact on social life. 112 patients were recruited: 43 osteoporotic and 69 cancer. 76 responses were obtained. Results: 112 cases were studied. 43 patients osteoporotic 69 oncological .74.1% women and 25.9% men. 76 responses were collected from the assigned surveys, as 30 patients died. Assigned scores were evaluated on a scale of 1 to 10 (1=very bad/10=not affected). Regarding the perceived symptoms, osteoporotic scored 4.18±2.8 compared to oncological 1.95±1.524. For the disruption of daily routine, osteoporotic scored 5.32±3.129 and oncological 3.24±2.823. Self-esteem was significantly higher in the osteoporotic: 4.63±3.657. The cure rate was significantly higher in the osteoporotic: 71.1% than in the oncological: 39.5%. Regarding the impact on social life, 76.3% of osteoporotic reported it as a problem, while 84.2% of oncological too. Conclusions: The addition of MRONJ to an underlying pathology can be counterproductive to the development of quality of life.

**INCIDENCE, CLINICAL OUTCOMES AND LONG-TERM SKELETAL RISK IN A COHORT OF PATIENTS WITH ATYPICAL FEMORAL FRACTURE**

**Loureyro MA, Quintero-Parga J, Kitaigrodsky AV, Aliquo N, Ullmann T, Buttazzoni M, Carabelli GS, Barla JD, Diehl M, Perez BM.**

*Hospital Italiano de Buenos Aires. Buenos Aires, Argentina.*

Atypical femoral fracture (AFF) is a rare but severe complication of prolonged bisphosphonate (BP) therapy. OBJECTIVE: To evaluate AFF incidence and subsequent fracture-free survival. METHODS: Retrospective cohort of AFF patients managed at a tertiary center (2014-2025). Incidence was calculated for individuals aged ≥50 within an institutional health system (2014-2023). Fragility fracture-free and subsequent AFF-free survival were analyzed using Kaplan-Meier curves. Longitudinal serum bCTX levels were assessed across different osteoactive regimens. RESULTS: 63 women with 69 AFFs were included (median age 77 years, IQR 70–83); all had prior BP exposure (median 93 months, IQR 60–132); 48% had bilateral involvement. The global incidence rate of complete AFF was 5.5 (95%CI 4.1–7.1) per 100,000 person-years. Over a median follow-up of 4.5 years (IQR 1.5–8.1), 18 fragility fractures (29%) and 10 deaths (16%) occurred. Four subsequent AFFs occurred among 53 patients at risk (7.6%; 95%CI 3-18%). Pre-AFF bCTX was 0.28 ng/mL (IQR 0.22–0.41). During follow-up (271 measurements in 56 patients), median bCTX varied by therapy: 0.46 ng/mL under teriparatide and 0.30 ng/mL under BPs. Fine-Gray analysis revealed a clinically relevant cumulative incidence or fragility fractures. Exploratory models identified no statistically significant predictors for new events of fracture of AFF. Plot analysis showed bCTX >1 ng/mL clustered near fragility fracture occurrences. CONCLUSIONS: This 10-year cohort demonstrates an AFF incidence of 5.5/100,000 women-years and a high subsequent fragility fracture burden, highlighting the need for active skeletal management post-AFF.

### UTILITY OF TRABECULAR BONE SCORE FOR IDENTIFYING VERTEBRAL FRACTURES IN RHEUMATOID ARTHRITIS: EVIDENCE FROM AN ARGENTINE COHORT

**Ramírez Stieben LA (1,2), Nasazzi Doddi P (2), Lombarte M (1,3), Brun LR (1,3), Brance ML (1,2,3)**

*(1) Bone Biology Laboratory, Faculty of Medicine, Rosario National University, Argentina. (2) Reumatología y Enfermedades Óseas Rosario, Argentina. (3) National Council of Scientific and Technical Research (CONICET), CCT-Rosario, Argentina.*

Introduction: Rheumatoid arthritis (RA) is associated with an increased risk of fragility fractures. Vertebral fractures are frequently underdiagnosed and have relevant clinical impact. Trabecular Bone Score (TBS), bone mineral density (BMD), and FRAX are used to assess fracture risk, but their relative utility for identifying prevalent vertebral fractures in RA remains incompletely defined. Materials and methods: Cross-sectional analytical study in adult patients with RA (2010 ACR/EULAR criteria) with available clinical data, DXA, TBS, DXA-based vertebral fracture assessment (VFA), and FRAX estimation using the Argentina-specific model. Vertebral fractures were semiquantitatively graded by VFA from T4 to L4. The primary outcome was the presence of at least one moderate/severe morphometric vertebral fracture. Multivariable logistic regression models were built in two families: without FRAX (Family A, clinical base model with sequential addition of lumbar BMD and/or TBS) and with FRAX major osteoporotic fracture probability as the main predictor (Family B, with sequential addition of lumbar BMD and/or TBS). Discrimination was assessed using ROC curves and AUC, compared with DeLong's test. Results: Ninety-three patients were included; 82% women, mean age 51±13 years, 63% postmenopausal, median disease duration 6 years [2–14], and mean DAS28 3.70±1.59. Mean TBS was 1.37±0.13; mean lumbar BMD 0.93±0.14 g/cm$^2$; median FRAX major osteoporotic fracture probability 5% [3–10]. Prevalent morphometric vertebral fractures were identified in 47/93 patients (50.5%); 31.2% had one fracture, 9.7% two, and 16.2% three or more. No significant differences were observed between groups in TBS, femoral BMD, or FRAX-derived probabilities; lumbar BMD was numerically lower in patients with fractures (0.91 vs 0.96 g/cm$^2$; p=0.10). In univariable analysis, age was associated with moderate/severe fractures (OR 1.04; 95%CI 1.00–1.09; p=0.040) and DAS28 showed a borderline association (OR 1.45; 95%CI 0.98–2.12; p=0.060). In adjusted models, neither TBS nor lumbar BMD independently predicted the outcome. In the sensitivity model, DAS28 was the only significantly associated variable (p=0.033). Discrimination was modest across all models (maximum AUC 0.639), and the addition of TBS did not significantly improve AUC (DeLong's test, p≥0.201). Conclusion: In this RA cohort, vertebral fracture burden was high. However, TBS did not provide incremental discriminative value beyond clinical variables, BMD, or FRAX. These findings suggest that vertebral fragility in RA may be more closely related to overall disease burden than to isolated densitometric or texture-derived indices.

The Congress organizers wish to gratefully acknowledge the following sponsors for their generous support of this meeting.

**ACADEMIC AND SCIENTIFIC SPONSORS:**

Asociación Argentina para el estudio del Climaterio
Facultad de Ciencias Exactas, Universidad Nacional de La Plata
Federación Argentina de Sociedades de Endocrinología
Instituto de Investigaciones Metabólicas
International Osteoporosis Foundation
Sanatorio Julio Méndez
Sociedad Argentina de Andrología
Sociedad Argentina de Nefrología
Sociedad de Endocrinología y Metabolismo de Córdoba
Sociedad Uruguaya de Endocrinología y Metabolismo

**SPONSORING COMPANIES:**

ACCESS MEDICAL SYSTEMS
ADIUM
AstraZeneca
BIOSIDUS
El Panta
ELEA
GADOR
Roemmers
TRB-Pharma
ULTRAGENYX
